# MdBBX21, a B-Box Protein, Positively Regulates Light-Induced Anthocyanin Accumulation in Apple Peel

**DOI:** 10.3389/fpls.2021.774446

**Published:** 2021-11-12

**Authors:** Bo Zhang, Zhen-Zhen Zhu, Dong Qu, Bo-Chen Wang, Ni-Ni Hao, Ya-Zhou Yang, Hui-Juan Yang, Zheng-Yang Zhao

**Affiliations:** ^1^State Key Laboratory of Crop Stress Biology for Arid Areas, College of Horticulture, Northwest A&F University, Yangling, China; ^2^Shaanxi Research Center of Apple Engineering and Technology, Yangling, China; ^3^Shaanxi Key Laboratory Bio-resources, College of Bioscience and Engineering, Shaanxi University of Technology, Hanzhong, China

**Keywords:** apple, light, anthocyanin, MdBBX21, MdMYB1

## Abstract

The red coloration of apple (*Malus* × *domestica* Borkh.) is due to the accumulation of anthocyanins in the fruit peel. Light is essential for anthocyanin biosynthesis in apple. In this study, we performed a transcriptome sequencing (RNA-seq) analysis of apple fruit exposed to light after unbagging. The identified differentially expressed genes included *MdBBX21*, which is homologous to Arabidopsis *BBX21*, suggesting it may be involved in light-induced anthocyanin biosynthesis. Additionally, MdBBX21 was localized in the nucleus and its gene was expressed earlier than *MdMYB1* in apple peel treated with light. Overexpressing *MdBBX21* in Arabidopsis and apple calli under light increased anthocyanin accumulation. Dual-luciferase and yeast one-hybrid assays confirmed that MdBBX21 binds to the *MdHY5*, *MdBBX20*, and *MdBBX22*-*1*/*2* promoters and induces expression. At the same time, MdHY5 can also activate the expression of *MdBBX21*. Furthermore, bimolecular fluorescence complementation and yeast two-hybrid assays demonstrated that MdBBX21 can interact with MdHY5. This interaction can significantly enhance *MdMYB1* promoter activity. These findings clarify the molecular mechanism by which MdBBX21 positively regulates light-induced anthocyanin accumulation in apple.

## Introduction

Apples are one of the most widely cultivated fruits worldwide. Red-skinned apples are more popular with consumers than green-skinned or yellow-skinned apples. The redness of the peel is mainly determined by anthocyanins, which are natural water-soluble pigments (Kim et al., 2003; Zhang Y. et al., 2014). Under natural conditions, anthocyanins are stored in plant vacuoles in the form of glycosides (Passeri et al., 2016). The diversity in the colors of flowers, stems, leaves, fruits, and other plant organs and tissues is due to the type, content, and distribution of anthocyanins (Lawrence et al., 1939; Zhang H. et al., 2014).

Anthocyanins are produced by the flavonoid pathway in a process that is controlled by a series of structural genes and regulatory factors (Winkel-Shirley, 2001). The structural genes (e.g., early and late biosynthetic genes) encode various enzymes in the plant anthocyanin biosynthetic pathway. The early biosynthetic genes include those encoding chalcone synthase (CHS), chalcone isomerase (CHI), and flavanone 3-hydroxylase (F3H), whereas the late biosynthetic genes include those encoding dihydroflavonol 4-reductase (DFR), anthocyanidin synthase (ANS), and flavonoid 3-O-glycosyltransferase (UFGT) (Martin et al., 1991; Kubasek et al., 1992). Transcriptional regulators control the expression of structural genes (Quattrocchio et al., 1993, 1998; de Vetten et al., 1997). For example, MYB proteins, which are among the most important transcription factors, can combine with bHLH and WD40 proteins to form an MBW complex that binds to the promoter of structural genes to induce expression and regulate anthocyanin synthesis (Ramsay and Glover, 2005; Li, 2014). In apple, MdMYB1 and MdMYBA are responsible for apple skin coloration (Takos A. M. et al., 2006; Ban et al., 2007). Five direct tandem repeats of the MdMYB10-binding motif in the *MdMYB10* promoter are associated with the accumulation of anthocyanins throughout the plant, ultimately resulting in striking phenotypes (e.g., red fruit flesh and red foliage) (Espley et al., 2007, 2009). By aligning the *MdMYB1* sequence with the GDDH13 and HFTH1 genomes, a long terminal repeat retrotransposon associated with the red-skinned phenotype was detected upstream of *MdMYB1* (Zhang et al., 2019). These results strongly indicate that the *MdMYB1/A/10* alleles are the core transcriptional regulatory genes of the anthocyanin biosynthesis pathway.

In addition to being genetically regulated, anthocyanin production is also affected by various environmental factors, including drought (An et al., 2020), low temperatures (Xie et al., 2012), salt stress (Lotkowska et al., 2015), low nitrogen availability (Sun et al., 2018), and light (Cominelli et al., 2008). For the biosynthesis of anthocyanins in the apple peel, light is the most important environmental factor (Merzlyak and Chivkunova, 2000). The mechanism underlying light-induced anthocyanin synthesis has been well characterized as part of photomorphogenesis in the model plant Arabidopsis (Maier et al., 2013). Light signals are perceived by plants and then transmitted by photoreceptor proteins such as phytochrome (PHY) (Sharrock and Quail, 1989), cryptochrome (CRY) (Lin et al., 1995), phototropin (PHOT) (Kasahara et al., 2002), and ultraviolet light receptor (UVR8) (Rizzini et al., 2011). Different photoreceptor proteins can perceive and transmit light of different wavelengths. The blue light receptor gene *MdCRY2* and the UV-B photoreceptor gene *MdUVR8* have been characterized in apple (Li et al., 2013; Zhao et al., 2016). Downstream of the photoreceptors, LONG HYPOCOTYL 5 (HY5), ubiquitin E3 ligase constitutive photobiochemical enzyme 1 (COP1), and PHYTOCHROME-INTERACTING FACTORS (PIFs) are involved in the complex signaling network mediating the regulation of plant photomorphogenesis (Deng et al., 1992; Ang and Deng, 1994; Ni et al., 1998). In apple, MdHY5 binds directly to the *MdMYB1* promoter to induce expression and promote the accumulation of anthocyanins under light conditions (An et al., 2017). However, under dark conditions, MdCOP1 can interact with MdMYB1 to mediate the ubiquitination and degradation of MdMYB1 (Li et al., 2012).

The B-box (BBX) protein is a zinc finger protein containing one or two B-box motifs (Reymond et al., 2001). It is a transcription factor that regulates plant photomorphogenesis along with HY5, COP1, and PIFs. Members of the BBX family have been identified in many species, including Arabidopsis (Khanna et al., 2009), rice (Huang et al., 2012), tomato (Chu et al., 2016), apple (Liu et al., 2018), and pear (Bai et al., 2019a). In Arabidopsis, the BBX protein family is divided into five subfamilies according to the protein structures. Subfamily IV comprises eight members (BBX18–25), each with two B-box zinc finger motifs (Khanna et al., 2009). The transcription factors in this subfamily are closely related to the light signaling cascade. They can interact with the COP1 and HY5 transcription factors, and can link the light signal regulatory network with other regulatory networks (e.g., hormone and temperature) (Sarmiento, 2013; Song et al., 2020). In apple, BBX22 positively regulates UV-B-induced anthocyanin synthesis, but its function depends on the synergistic effect of HY5 (Bai et al., 2014; An et al., 2019). Additionally, MdBBX20 can form a complex with MdHY5 and bind directly to the *MdMYB1*, *MdDFR*, and *MdANS* promoters to promote anthocyanin synthesis in apple (Fang et al., 2019b). Moreover, MdCOL4 interacts with MdHY5 to inhibit the expression of *MdMYB1*, whereas it can bind directly to the *MdUFGT* and *MdANS* promoters to suppress expression (Fang et al., 2019a). However, whether there are other BBX proteins involved in the synthesis of anthocyanins in apple and the relationships among these BBX proteins remain unclear.

Although the mechanism mediating light-induced anthocyanin synthesis has been partially characterized, a thorough analysis is still needed. In this study, on the basis of transcriptome sequencing data, we revealed that *MdBBX21* is differentially expressed in the peel when unbagged fruit are exposed to light. Additionally, *MdBBX21* responds to light and reaches peak expression levels earlier than *MdBBX20*, *MdBBX22*-*1*/*2*, and *MdHY5*. The overexpression of *MdBBX21* in transgenic Arabidopsis plants and apple calli can induce anthocyanin accumulation under light. Further analyses indicated that MdBBX21 can activate the expression of *MdBBX20*, *MdBBX22*-*1*/*2*, and *MdHY5*, which subsequently promotes anthocyanin accumulation. Furthermore, MdBBX21 can interact with MdHY5 and induce the expression of *MdMYB1*. These findings clarify the transcriptional regulation that occurs upstream of MdMYB1 during light-induced anthocyanin biosynthesis.

## Materials and Methods

### Plant Materials

Six-year-old “Starkrimson Delicious” apple trees growing at the Baishui Apple Experimental Farm of Northwest A&F University, Shaanxi province, China were used as experimental materials. Fruit were covered with a paper bag (Hongtai, Shanxi, China) at 45 days after blooming and harvested at 135 days after blooming. The harvested fruit were placed in an incubator at 23°C under continuous white light (200 μmol m^–2^ s^–1^). The peels were excised at 0, 3, 6, 9, 12, 24, 48, and 72 h after initiating the light treatment. Twelve fruit were selected at each time-point, with four fruit considered as one biological replicate.

### Determination of the Anthocyanin Content

Anthocyanins were extracted from the apple peels as previously described (Xie et al., 2012). Briefly, 0.5 g peel was treated with 5 mL 1% (v/v) HCl-methanol and incubated in darkness at 4°C for 24 h. After centrifuging at 13,000 × *g* for 10 min, the anthocyanin content in the upper liquid layer was determined using an HPLC system comprising a Waters 2998 detector (Waters, Milford, MA, United States) and a C18 column (5 μm internal diameter, 250 mm × 4.6 mm; Waters) as previously described (Liu et al., 2013). The anthocyanin content of Arabidopsis was determined as previously described (Wang et al., 2016).

### RNA Extraction, Library Preparation, and RNA-seq

Total RNA was extracted using the TRIzol RNA Plant Plus Reagent (Tiangen, Beijing, China). The integrity of the RNA was checked using the 2100 Bioanalyzer (Agilent Technologies, Palo Alto, CA, United States) and by agarose gel electrophoresis. The purity of the RNA was determined using the NanoPhotometer spectrophotometer (IMPLEN, Westlake Village, CA, United States). Sequencing libraries were constructed using the NEBNext^®^ Ultra^*TM*^ RNA Library Prep Kit for Illumina (NE, United States). After verifying the quality of the libraries, they were sequenced using the Illumina Novaseq 6000 sequencer (150-bp paired-end sequencing) (Illumina, San Diego, CA, United States) at Novogene Bioinformatics Technology Co., Ltd., Beijing, China.

After eliminating the reads with sequencing adapters and the low-quality reads from the raw data, the remaining clean reads were aligned to the *Malus* Genome GDDH13 reference sequence (version 1.1) (Daccord et al., 2017) using the HISAT2 software. After the sequence alignment, the raw counts of the mapped reads for each *Malus* gene model in GDDH13 (version 1.1) were determined and then normalized to FPKM per million mapped reads. Using DESeq2, differentially expressed genes (DEGs) were putatively identified according to the following criteria: | log_2_(fold-change)| > 1 and adjusted padj < 0.05.

### Subcellular Localization

The *MdBBX21* coding sequence (CDS) was cloned from the cDNA derived from “Starkrimson Delicious” fruit peel. The full-length *MdBBX21* open reading frame without the stop codon was inserted into the pCAMBIA2301-eGFP vector under the control of the CaMV 35S promoter to obtain the 35S:MdBBX21-eGFP construct. The primer sequences used are listed in Supplementary Table 4. Tobacco (*Nicotiana benthamiana*) leaves were infiltrated with *Agrobacterium tumefaciens* strain GV3101 cells carrying 35S:MdBBX21-eGFP or the empty vector control (pCAMBIA2301-eGFP). After 3 days, the eGFP signal in the tobacco leaves were detected using the LSM 710 confocal laser-scanning microscope (Carl Zeiss) at excitation wavelengths of 488 nm for eGFP.

### Analysis of mRNA Expression

First-strand cDNA was synthesized using the HiScript^®^ II Q RT SuperMix for qPCR (Vazyme, Nanjing, China). A quantitative real-time polymerase chain reaction (qPCR) analysis was conducted using the ChamQ Universal SYBR qPCR Master Mix (Vazyme) and the ABI StepOnePlus^*TM*^ Real-Time PCR System (Applied Biosystems, Waltham, MA, United States). The *MdActin* gene was used as the internal control. All expression data were examined according to the delta-delta cycle threshold method (Livak and Schmittgen, 2001). The primer sequences used are listed in Supplementary Table 4.

### Generation of Transgenic Plant Materials

“Orin” apple calli were transformed according to a slightly modified version of a previously reported method (Xie et al., 2012). First, calli were infected with *A. tumefaciens* strain LBA4404 cells carrying 35S:MdBBX21-eGFP for 20 min. The calli were then transferred to MS medium supplemented with 1 mg L^–1^ 6-benzylaminopurine (6-BA), 1 mg L^–1^ 2,4-dichlorophenoxyacetic acid (2,4-D), 30 g L^–1^ sucrose, and 8 g L^–1^ agar. After a 3-day incubation in darkness, the calli were transferred to screening medium (i.e., MS medium supplemented with 30 g L^–1^ sucrose, 1 mg L^–1^ 2,4-D, 1 mg L^–1^ 6-BA, 8 g L^–1^ agar, 250 mg L^–1^ carbenicillin, and 50 mg L^–1^ kanamycin) to screen for transformants. For the light treatment, wild-type (WT) and *MdBBX21*-overexpressing (MdBBX21-OX) transgenic apple calli cultivated in darkness were transferred to a light incubator and placed under constant white light (500 μmol m^–2^ s^–1^) for 3 days.

*Agrobacterium tumefaciens* strain GV3101 cells carrying 35S:MdBBX21-eGFP were used to infect the Arabidopsis *bbx21* mutant (SALK_105390) according to the floral-dip method (Clough and Bent, 1998). Seeds of WT and T_3_ transgenic Arabidopsis plants were chilled at 4°C for 48 h and then placed under white light (500 μmol m^–2^ s^–1^) at 24°C under long-day conditions (16-h light/8-h dark). The anthocyanin contents of 5-day-old Arabidopsis seedlings were determined.

### Yeast Two-Hybrid Assay

A yeast two-hybrid assay was performed using the Matchmaker^*TM*^ Gold Yeast Two-Hybrid System (Clontech). The full-length *MdBBX21* CDS was inserted into the pGADT7 (AD) vector to construct the AD-BBX21 recombinant plasmid. The *MdHY5* CDS was inserted into the pGBKT7 (BD) vector to obtain the BD-HY5 recombinant plasmid. The primer sequences used are listed in Supplementary Table 4. Yeast strain Y2HGold cells were transformed with AD-BBX21 + BD-HY5, AD + BD-HY5, AD-BBX21 + BD, pGADT7-T + pGBKT7-53, or pGADT7-T + pGBKT7-Lam and then cultured on medium lacking tryptophan and leucine at 30°C. To screen for interacting proteins, the yeast cells were transferred to medium lacking leucine, tryptophan, histidine, and adenine (−L/−T/−H/−A), but supplemented with X-α-gal.

### Yeast One-Hybrid Assay

The *MdBBX20*, *MdBBX22*-*1*/*2*, *MdHY5*, and *MdMYB1* promoter fragments were inserted into the pHIS2 vector to construct the MdBBX20pro-HIS2, MdBBX22-1pro-HIS2, MdBBX22-2pro-HIS2, MdHY5pro-HIS2, and MdMYB1pro-HIS2 recombinant plasmids. The primer sequences used are listed in Supplementary Table 4. To determine the optimal 3-AT concentration, yeast strain Y187 cells containing the recombinant pHIS2 plasmids were grown on screening medium lacking histidine and tryptophan (−H/−T), but supplemented with different 3-AT concentrations. Next, Y187 yeast cells were co-transformed with MdBBX21-AD and individual recombinant pHIS2 plasmids. Interactions were detected on selection medium lacking histidine, tryptophan, and leucine (−H/−T/−L), but supplemented with the optimal 3-AT concentration.

### Transient Dual-Luciferase Assay

Transient dual-luciferase assays were performed using tobacco (*N. benthamiana*) leaves. The *MdBBX20*, *MdBBX22*-*1*/*2*, *MdHY5*, and *MdMYB1* promoter fragments were inserted into the pGreenII 0800-LUC vector to construct the MdBBX20pro:LUC, MdBBX22-1pro:LUC, MdBBX22-2pro:LUC, MdHY5pro:LUC, and MdMYB1pro:LUC recombinant plasmids. The full-length *MdBBX21* CDS was inserted into the pGreenII 62-SK vector. The primer sequences used are listed in Supplementary Table 4. *A. tumefaciens* strain GV3101 cells carrying the pSoup vector were transformed with the recombinant plasmids. Leaves from 5-week-old tobacco (*N. benthamiana*) plants were injected according to a previously described method (Zhang et al., 2020). In order to maintain the same concentration of *Agrobacterium* solution, 50 ul of *Agrobacterium* solution MdBBX21 (OD = 0.8) and MdHY5 (OD = 0.8) were mixed with 50 ul of *Agrobacterium* solution MdMYB1pro:LUC for tobacco leaf injection, respectively. Also, equal volumes and concentrations of *Agrobacterium* solution MdBBX21 and MdHY5 were mixed, and then 50 ul of the mixture was mixed with 50 ul of *Agrobacterium* solution MdMYB1pro:LUC for tobacco leaf injection.

### Bimolecular Fluorescence Complementation Assay

The *MdBBX21* CDS was inserted into the pSPYNE vector, whereas the *MdHY5* CDS was inserted into the pSPYCE vector. *A. tumefaciens* strain GV3101 cells were transformed with the MdBBX21-NE and MdHY5-CE recombinant plasmids. The primer sequences used are listed in Supplementary Table 4. Equal volumes of the *A. tumefaciens* cells carrying MdBBX21-NE and MdHY5-CE were mixed. Arabidopsis protoplast cells were infected with the *A. tumefaciens* cells for 15 min and then incubated at 23°C for 16–20 h. The YFP fluorescence was detected using the LSM 710 confocal laser-scanning microscope (Carl Zeiss) with an excitation wavelength of 514 nm.

## Results

### Transcriptome Analysis of Apple Peel Exposed to Light

**FIGURE 1:**
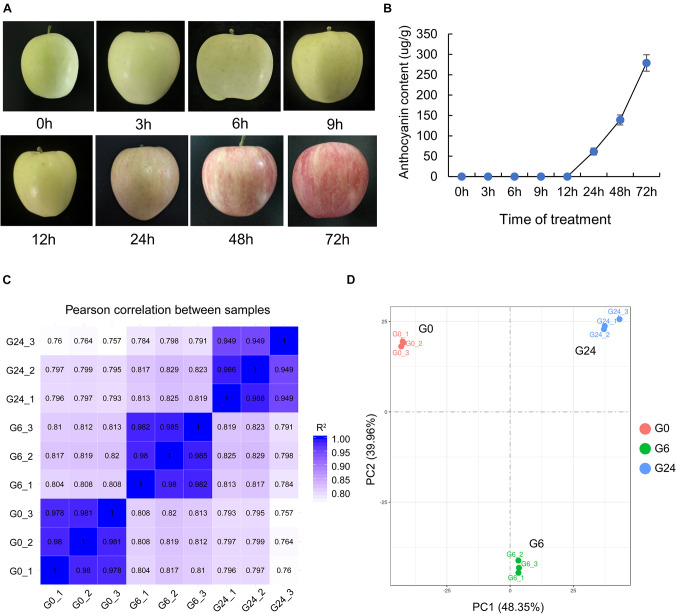
RNA-seq data of “Starkrimson Delicious” apple fruit exposed to light after unbagging. **(A)** Change in color of “Starkrimson Delicious” fruit peel under light. **(B)** Anthocyanin content in the peel under light. **(C)** Pearson correlation between samples. **(D)** Principal component analysis of the RNA-seq data.

To clarify the response of apple to light, we first measured the anthocyanin content in the peel of unbagged apple fruit exposed to white light for 0, 3, 6, 9, 12, 24, 48, and 72 h (Figure 1A). Anthocyanins were basically undetectable before 12 h, but they started to accumulate significantly in the peel at 24 h (Figure 1B). The peel samples at the following three time-points underwent an RNA-seq analysis: 0 h (G0), 6 h (G6), and 24 h (G24). After the strict data filtering step, the number of clean reads in each library ranged from 37.69 to 45.95 million, and the Q30 values exceeded 92% (Supplementary Table 1). The mapping rate of the clean reads to the reference genome was 91.25–92.51%. Additionally, 88.81–90.27% of the clean reads were uniquely mapped (Supplementary Table 2). All biological replicates were strongly correlated (*R*^2^ > 0.94) (Figure 1C); this correlation was confirmed by principal component analysis (Figure 1D).

### Identification of Differentially Expressed Genes and Kyoto Encyclopedia of Genes and Genomes Analysis

**FIGURE 2:**
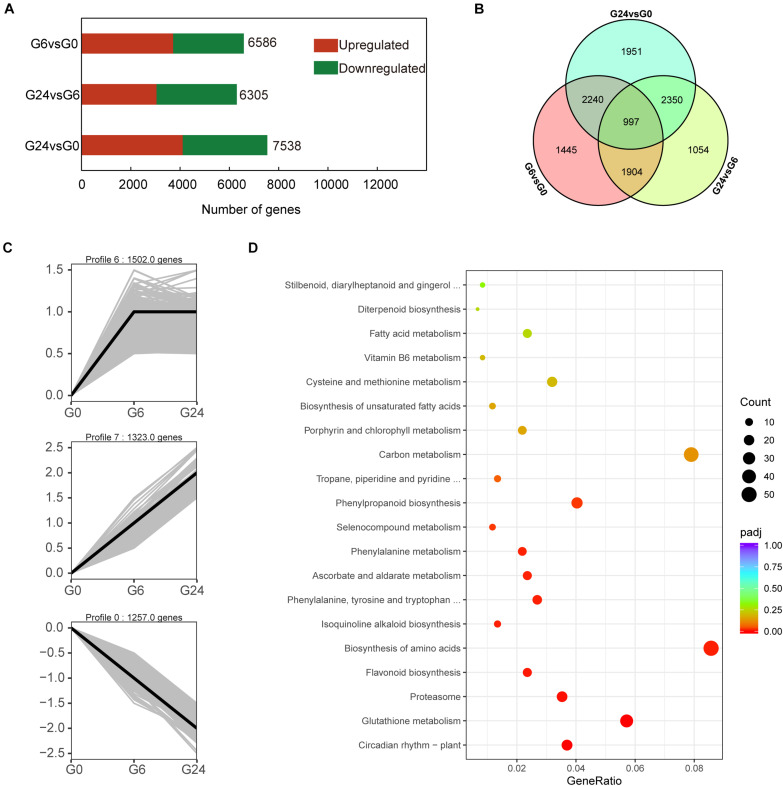
Identification of DEGs and KEGG analysis. **(A)** Number of DEGs between samples. **(B)** Venn diagram representation of DEGs from pairwise comparisons. **(C)** Three significant gene expression profiles. **(D)** KEGG pathway enrichment analysis of differentially expressed transcripts in profiles 0, 6, and 7.

To screen for early light-responsive genes, we compared the G0, G6, and G24 expression levels. In total, 11,941 DEGs were revealed in the G6 vs. G0, G24 vs. G6, and G24 vs. G0 pairwise comparisons. More DEGs were detected in the G6 vs. G0 comparison than in the G24 vs. G6 comparison (Figures 2A,B). These results suggest that many genes started to respond to light signals after 6 h. To further elucidate the gene expression patterns, the 11,941 DEGs were classified into eight gene expression profiles (Supplementary Figure 1). Specifically, 4,082 DEGs were classified into three significant profiles (*P* < 0.05), including two up-regulated profiles (profiles 6 and 7) and one down-regulated profile (profile 0) (Figure 2C). A KEGG enrichment analysis indicated genes related to flavonoid biosynthesis and phenylpropanoid biosynthesis were significantly enriched among the DEGs in profiles 0, 6, and 7 (Figure 2D).

### Characterization and Expression Analysis of MdBBX21

**FIGURE 3:**
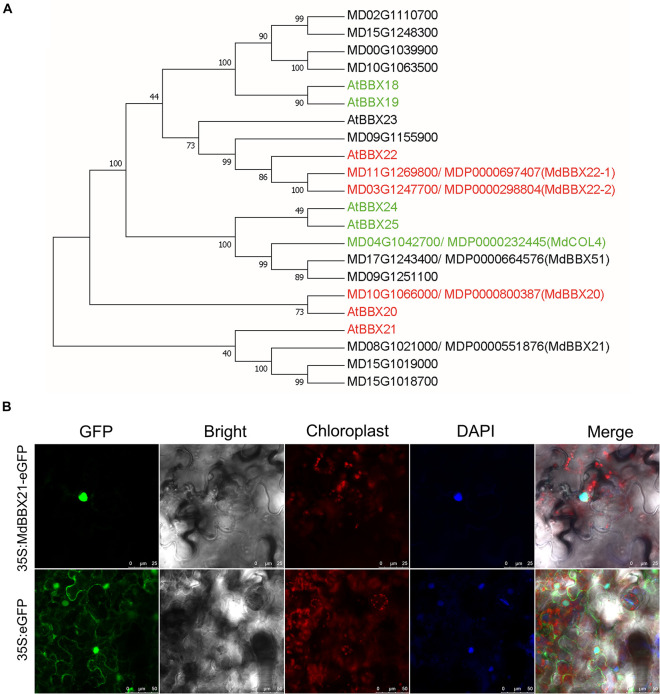
Characterization of *MdBBX21*. **(A)** Phylogenetic relationship of apple and Arabidopsis subfamily IV members. The red and green genes represent positive and negative regulators related to light signaling, respectively. The phylogenetic tree was constructed using the neighbor-joining method by MEGA 7.0. The bootstrap values of 1000 replicates were calculated at each node. **(B)** Subcellular localization of MdBBX21 expressed in tobacco leaf cells.

Among the DEGs in up-regulated profile 6 (Supplementary Table 3), *MD08G1021000* was revealed to be highly homologous to Arabidopsis *AtBBX21*. Thus, we named this gene *MdBBX21*. To clarify the relationship between MdBBX21 and MdBBX20, MdBBX21, and MdBBX22-1/2, we compared their amino acid sequences. The sequence identities between MdBBX21 and MdBBX20, MdBBX22-1, and MdBBX22-2 were 37.58, 31.92, and 31.63%, respectively. In the constructed phylogenetic tree, MdBBX21, MdBBX20, and MdBBX22-1/2 were clustered with Arabidopsis subfamily IV members, but they were distributed in different clades (Figure 3A).

The correct cellular localization of a protein is critical for ensuring it functions properly. In tobacco leaf cells transiently transformed with 35S:MdBBX21-eGFP, green fluorescence was detected only in the nucleus, whereas in the control tobacco cells transiently transformed with 35S:eGFP, green fluorescence was observed throughout the cell, including in the cytoplasm and nucleus. Accordingly, MdBBX21 appears to be a nuclear protein (Figure 3B).

**FIGURE 4:**
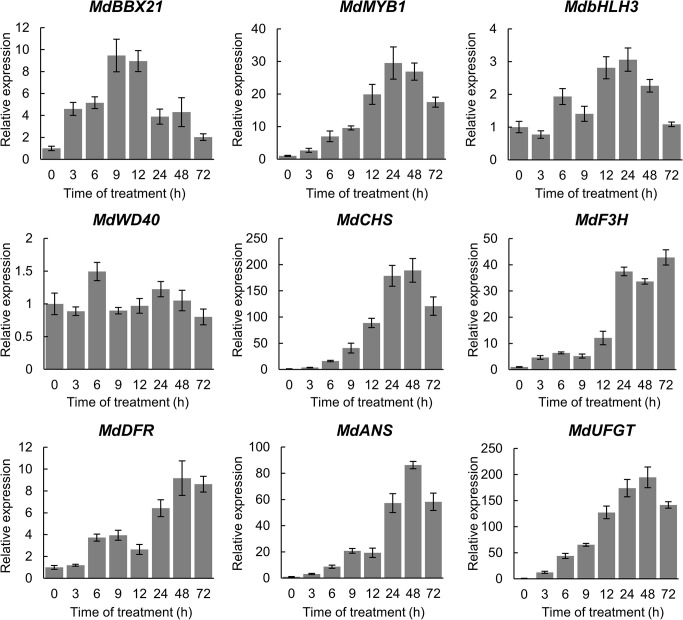
Relative expression levels of anthocyanin biosynthesis-related genes in the apple fruit peel irradiated with white light. The *MdActin* gene was used as the internal control.

To determine whether MdBBX21 affects anthocyanin synthesis, we analyzed *MdBBX21* expression in the apple fruit peel irradiated with white light for 0, 3, 6, 9, 12, 24, 48, and 72 h. The *MdBBX21* gene was responsive to light, and its expression level started to increase at 3 h, peaking at 9 h. The expression of the regulatory genes (*MdMYB1* and *MdbHLH3*) and structural genes (*MdCHS*, *MdF3H*, *MdDFR*, *MdANS*, and *MdUFGT*) related to anthocyanin synthesis peaked after 24 h. The *MdWD40* expression level was essentially unchanged (Figure 4). These results imply that MdBBX21 may function upstream of MdMYB1.

### Heterologous Overexpression of MdBBX21 Promotes Anthocyanin Accumulation in Arabidopsis

**FIGURE 5:**
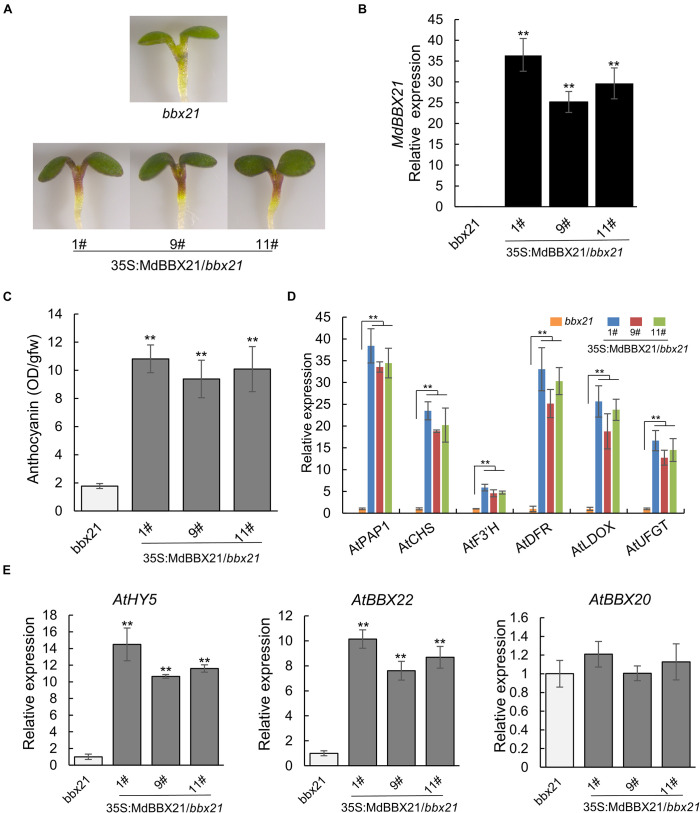
Overexpression of *MdBBX21* promotes anthocyanin accumulation in Arabidopsis seedlings. **(A)** Phenotypes of 35S:MdBBX21/*bbx21* and *bbx21* mutant seedlings. 35S:MdBBX21/*bbx21* (#1), 35S:MdBBX21/*bbx21* (#9), and 35S:MdBBX21/*bbx 21* (#11) represent three transgenic lines. **(B)** The *MdBBX21*’s expression level in 35S:MdBBX21/*bbx21* and *bbx21* mutant seedlings. **(C)** Anthocyanin content in 35S:MdBBX21/*bbx21* and *bbx21* mutant seedlings. **(D)** Relative expression levels of anthocyanin biosynthesis-related genes in 35S:MdBBX21/*bbx21* and *bbx21* mutant seedlings. **(E)** Relative expression levels of *AtHY5*, *AtBBX22*, and *AtBBX20* in 35S:MdBBX21/*bbx21* and *bbx21* mutant seedlings. Error bars represent the standard deviation of three biological replicates. **P < 0.01 (Student’s *t*-test).

To confirm MdBBX21 contributes to anthocyanin biosynthesis, *MdBBX21* was overexpressed in the *bbx21* Arabidopsis mutant. The 35S:MdBBX21/*bbx21* line accumulated more anthocyanins in the cotyledons and hypocotyls than the untransformed *bbx21* Arabidopsis mutant (Figures 5A–C). The expression levels of anthocyanin-related genes (*AtPAP1*, *AtCHS*, *AtF3’H*, *AtDFR*, *AtLDOX*, and *AtUFGT*) also increased in the 35S:MdBBX21/*bbx21* line, which was consistent with the changes in the anthocyanin content (Figure 5D). We also examined the expression of *AtHY5* and Arabidopsis *BBX* subfamily IV members. The *AtHY5* and *AtBBX22* expression levels were higher in the 35S:MdBBX21/*bbx21* seedlings than in the *bbx21* Arabidopsis mutant seedlings. In contrast, there were no differences in the expression levels of the other subfamily IV *BBX* genes (Figure 5E and Supplementary Figure 2).

### Overexpression of MdBBX21 Promotes Anthocyanin Accumulation in Apple Calli

**FIGURE 6:**
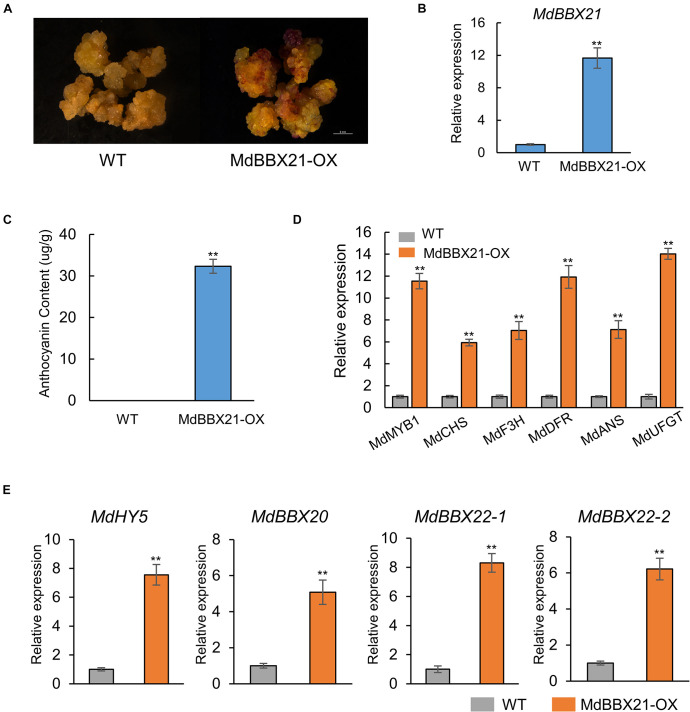
Overexpression of *MdBBX21* promotes anthocyanin accumulation in apple calli. **(A)** Anthocyanin accumulation phenotypes of apple calli (WT: wild-type apple calli; MdBBX21-OX: *MdBBX21* overexpression apple calli). **(B)** *MdBBX21*’s expression level in WT and MdBBX21-OX. **(C)** Anthocyanin contents in WT and MdBBX21-OX. **(D)** The expression levels of anthocyanin biosynthesis-related genes in WT and MdBBX21-OX. **(E)** The expression levels of *MdHY5*, *MdBBX20*, and *MdBBX22*-*1*/*2* in WT and MdBBX21-OX. Error bars represent the standard deviation of three biological replicates. **P < 0.01 (Student’s *t*-test).

To further confirm the MdBBX21 function related to anthocyanin biosynthesis in apple, we overexpressed *MdBBX21* in apple calli (Figures 6A,B). Wild-type and MdBBX21-OX transgenic apple calli were cultured on medium in darkness and then transferred to a light incubator for a 3-day exposure to constant light. The MdBBX21-OX apple calli accumulated more anthocyanins than the WT calli (Figure 6C). As expected, the expression levels of the anthocyanin biosynthesis-related genes (*MdMYB1*, *MdCHS*, *MdF3H*, *MdDFR*, *MdANS*, and *MdUFGT*) were significantly higher in the MdBBX21-OX calli than in the WT calli (Figure 6D). These results suggest that MdBBX21 might positively regulate anthocyanin accumulation by promoting the transcription of anthocyanin biosynthesis-related genes. The transcription levels of the previously characterized genes *MdBBX20*, *MdBBX22*-*1*/*2*, *MdCOL4* (*MdBBX24*), and *MdHY5* were also analyzed by qPCR (Figure 6E and Supplementary Figure 3). The data indicated that *MdHY5*, *MdBBX20*, and *MdBBX22*-*1*/*2* were significantly more highly expressed in MdBBX21-OX calli than in WT calli. The *MdBBX24* expression level did not differ between the MdBBX21-OX and WT calli.

### MdBBX21 Binds Directly to the MdHY5, MdBBX20, and MdBBX22-1/2 Promoters and Induces Expression

**FIGURE 7:**
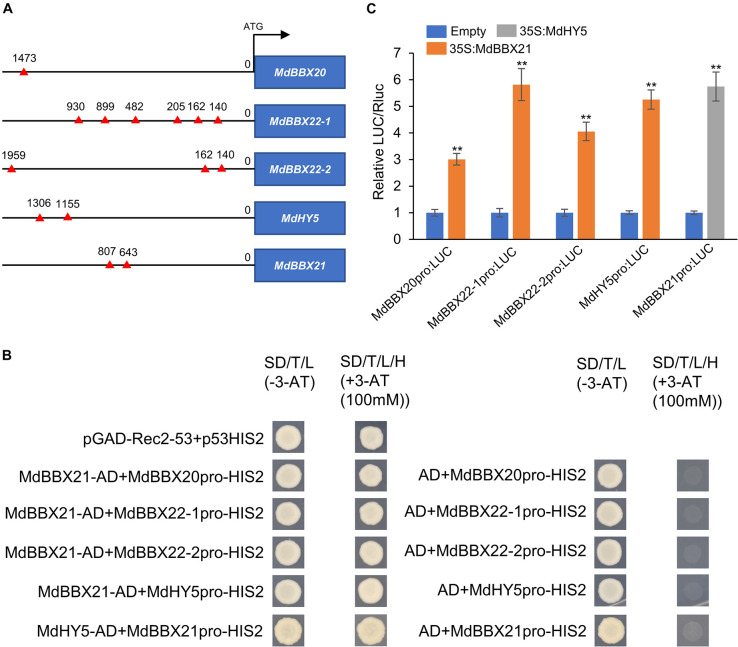
MdBBX21 binds directly to the *MdHY5*, *MdBBX20*, and *MdBBX22*-*1*/*2* promoters and induces expression. **(A)** The characteristics of G-box motif in the promoters of *MdHY5*, *MdBBX20*, and *MdBBX22*-*1*/*2*. The red triangle represents G-box motif. **(B)** Yeast one-hybrid assays identified interaction of MdBBX21 with the promoters of *MdHY5*, *MdBBX20*, and *MdBBX22*-*1*/*2*. **(C)** The effects of MdBBX21 on the promoter activity of *MdHY5*, *MdBBX20*, and *MdBBX22*-*1*/*2* with the dual-luciferase reporter assay. Error bars represent the standard deviation of three biological replicates. **P < 0.01 (Student’s *t*-test).

The increased *MdHY5*, *MdBBX20*, and *MdBBX22*-*1*/*2* expression levels in MdBBX21-OX calli led us to speculate that MdBBX21 may directly promote the expression of these four genes. Thus, we first searched for the G-box motif, which is a BBX-binding site (Datta et al., 2008; Xu et al., 2016), in the *MdHY5*, *MdBBX20*, and *MdBBX22*-*1*/*2* promoters. The G-box motif was detected in all three promoters (Figure 7A). In yeast one-hybrid assays, the Y187 yeast strains containing MdBBX21-AD + MdBBX20pro-HIS2, MdBBX21-AD + MdBBX22-1pro-HIS2, MdBBX21-AD + MdBBX22-2pro-HIS2, and MdBBX21-AD + MdHY5pro-HIS2 were able to grow on the −H/−T/−L selection medium containing 100 mM 3-AT. In contrast, the Y187 yeast strains containing AD + MdBBX20pro-HIS2, AD + MdBBX22-1pro-HIS2, AD + MdBBX22-2pro-HIS2, and AD + MdHY5pro-HIS2 did not grow under the same conditions (Figure 7B). This observation suggests that MdBBX21 can bind to the *MdHY5*, *MdBBX20*, and *MdBBX22*-*1*/*2* promoters. The dual-luciferase assay results confirmed that MdBBX21 can promote the transcription of *MdHY5*, *MdBBX20*, and *MdBBX22*-*1*/*2* (Figure 7C). We also analyzed the *MdHY5*, *MdBBX20*, and *MdBBX22*-*1*/*2* expression patterns in the peel exposed to light. Compared with the *MdBBX21* expression pattern (Figure 4), the *MdBBX20*, and *MdBBX22*-*1*/*2* expression levels increased significantly at 6 h, and their peak expression levels occurred later than that of *MdBBX21* (Supplementary Figure 4). These findings imply that MdBBX21 functions upstream of MdBBX20, and MdBBX22-1/2 and induces the expression of *MdBBX20*, and *MdBBX22*-*1*/*2*. At the same time, we found that *MdHY5* was activated at 3 h. Considering HY5 being a transcription factor, we speculated that MdHY5, a master regulator of light signaling, might also control the expression of *MdBBX21*. Thus, we searched for the G-box motif in the *MdBBX21* promoter. There were two G-box motifs present in the *MdBBX21* promoter (Figure 7A). Yeast one-hybrid assay results showed that MdHY5 can bind to the *MdBBX21* promoter fragment containing these two motifs (Figure 7B). The dual-luciferase assay results confirmed that MdHY5 can promote the transcription of *MdBBX21* (Figure 7C).

### The Interaction Between MdBBX21 and MdHY5 Can Significantly Enhance MdMYB1 Promoter Activity

**FIGURE 8:**
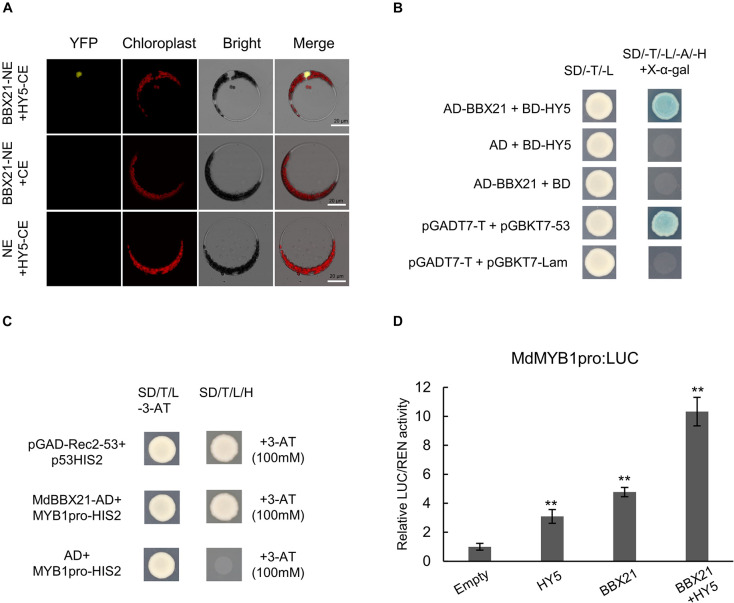
The interaction between MdBBX21 and MdHY5 can significantly enhance *MdMYB1* promoter activity. **(A)** Bimolecular fluorescence complementation assay. **(B)** Yeast two-hybrid assay showing interaction between MdBBX21 and MdHY5. **(C)** Yeast one-hybrid assay showing interaction between MdBBX21 and *MdMYB1* promoter. **(D)** Effects of MdBBX21 and MdHY5 individually and in combination on promoter activity of *MdMYB1* as determined by luciferase reporter assay. Error bars represent the standard deviation of three biological replicates. **P < 0.01 (Student’s *t*-test).

Because MdBBX20 (Fang et al., 2019b), MdBBX22-2 (An et al., 2019), PpBBX16 (Bai et al., 2019a), and PpBBX18 (Bai et al., 2019b) can interact with HY5, we speculated that MdBBX21 may also interact with MdHY5. Bimolecular fluorescence complementation assays were conducted to examine whether MdBBX21 can interact directly with MdHY5. Full-length *MdBBX21* and *MdHY5* CDSs were cloned into pSPYNE and pSPYCE vectors, respectively, to generate MdBBX21-NE and MdHY5-CE. Yellow fluorescence was detected in the nucleus of Arabidopsis protoplasts co-transformed with MdBBX21-NE and MdHY5-CE. However, yellow fluorescence was not observed in the BBX21-NE + CE and NE + HY5-CE controls (Figure 8A). These results indicate that MdBBX21 can interact with MdHY5. This interaction was verified in yeast two-hybrid assays (Figure 8B).

MdBBX20 can bind directly to the *MdMYB1* promoter and induce expression (Fang et al., 2019b). In this study, Y187 yeast strains containing MdBBX21-AD + MdMYB1pro-HIS2 grew on −H/−T/−L selection medium containing 100 mM 3-AT, but the Y187 yeast strains containing AD + MdMYB1pro-HIS2 did not (Figure 8C). Accordingly, MdBBX21 appears to be able to interact with the *MdMYB1* promoter. On the basis of the dual-luciferase assay results, MdBBX21 can promote *MdMYB1* expression (Figure 8D). Moreover, MdHY5 can also promote *MdMYB1* expression, which is consistent with the results of an earlier study (An et al., 2017). We also determined that the MdBBX21–MdHY5 heterodimer enhances the *MdMYB1* promoter activity more than MdBBX21 or MdHY5 alone (Figure 8D).

## Discussion

### MdBBX21 Responds to Light and Induces Anthocyanin Biosynthesis in Apple

Light is required for anthocyanin biosynthesis in the apple fruit peel (Saure, 1990). An exposure to light up-regulates the expression of anthocyanin biosynthesis-related structural and regulatory genes in the apple peel (Takos M. A. et al., 2006; Feng et al., 2013). However, how light signals affect the expression of these genes was unclear. There is increasing evidence that BBX proteins affect plant photomorphogenesis (Gangappa and Botto, 2014). The RNA-seq analysis in this study revealed that *MdBBX21* expression in the peel of dark-grown “Starkrimson Delicious” apples is affected by the subsequent exposure to light. Additionally, MdBBX21 and Arabidopsis AtBBX21 sequences are highly similar. Earlier research demonstrated that AtBBX21 is a positive regulator of anthocyanin biosynthesis (Datta et al., 2007). Hence, we predicted that MdBBX21 and AtBBX21 might have similar functions. We analyzed *MdBBX21* expression and observed that it increased in response to light. Moreover, *MdBBX21* expression peaked before the expression of anthocyanin-related structural and regulatory genes peaked (Figure 4). The overexpression of *MdBBX21* in the *bbx21* Arabidopsis mutant resulted in a significant increase in the anthocyanin contents of hypocotyls (Figure 5). This is consistent with the fact pear PpBBX18 (homologous to AtBBX21) regulates anthocyanin accumulation in transgenic Arabidopsis plants. In the present study, the MdBBX21-OX apple calli accumulated more anthocyanins than the WT apple calli under light (Figure 6). These results indicate that MdBBX21 is responsive to light and induces anthocyanin biosynthesis.

### MdBBX21 Up-Regulates MdBBX20, MdBBX22-1/2, and MdHY5 Expression

The Arabidopsis BBX subfamily IV members AtBBX20, AtBBX21, AtBBX22, and AtBBX23 positively regulate plant photomorphogenic processes in response to diverse light signals (Datta et al., 2007; Chang et al., 2008; Datta et al., 2008; Fan et al., 2012; Xu et al., 2016; Zhang et al., 2017). In pear, PpBBX16 and PpBBX18, which are subfamily IV BBX proteins, positively regulate anthocyanin synthesis by interacting with PpHY5 (Bai et al., 2019a,b). In apple, MdBBX20 and MdBBX22-1/2 reportedly promote anthocyanin accumulation in response to UV-B irradiation (Bai et al., 2014; An et al., 2019; Fang et al., 2019b). In addition, Plunkett et al. (2019) found that BBX subfamily IV member *MdBBX51* and BBX subfamily I member *MdBBX1* have very high expression levels during fruit development. Further, they can activate the promoter of *MdMYB1* in the presence of some co-factors MYB and bHLH. However, the relationships among these BBX proteins are unknown. In this study, *MdBBX21* expression increased significantly after a 3-h light treatment, and subsequently peaked at 9 h. In contrast, the *MdBBX20* and *MdBBX22*-*1*/*2* expression levels peaked at 12 and 24 h, respectively. The expression of structural and regulatory genes related to anthocyanin synthesis increased rapidly and peaked after 24 h (Figure 4 and Supplementary Figure 2). These findings indicate that MdBBX21 responds to light signals relatively early and likely functions upstream of MdBBX20 and MdBBX22-1/2. Dual-luciferase and yeast one-hybrid assays proved that MdBBX21 induces *MdBBX20* and *MdBBX22*-*1*/*2* expression after binding to their promoters. The *AtBBX22*, *MdBBX20*, and *MdBBX22*-*1*/*2* expression levels increased in Arabidopsis and apple calli overexpressing *MdBBX21* (Figures 5–7). Therefore, MdBBX21 can up-regulate the expression of *AtBBX22*, *MdBBX20*, and *MdBBX22*-*1*/*2*. However, MdBBX21 did not activate the expression of *MdBBX1*, *MdBBX24*, and *MdBBX51* in apple calli (Supplementary Figure 3). In Arabidopsis, AtBBX21 can bind directly to the *AtHY5* promoter and induce expression (Xu et al., 2016). In the current study, biochemical assays proved that MdBBX21 can bind directly to the *MdHY5* promoter and induce expression (Figure 7). The overexpression of *MdBBX21* in Arabidopsis and apple calli resulted in up-regulated *HY5* expression (Figures 5, 6). These results imply that MdBBX21 and AtBBX21 have similar functions and can directly up-regulate the expression of *HY5*. At the same time, we found that MdHY5 can also activate the expression of *MdBBX21*, indicating that a positive feedback regulation mechanism exists between MdBBX21 and MdHY5.

### The MdBBX21–MdHY5 Interaction Can Significantly Enhance MdMYB1 Promoter Activity Under Light

The bZIP transcription factor AtHY5 is a positive regulator of plant photomorphogenesis that functions downstream of the photoreceptors and COP1 (Lau and Deng, 2010). Additionally, AtHY5 regulates various physiological processes, including anthocyanin synthesis, lateral root formation, and hypocotyl elongation (Oyama et al., 1997). Earlier studies regarding *in vitro* DNA–protein interactions demonstrated that AtHY5 can bind directly and specifically to the G-box motif in the promoters of the anthocyanin-related structural genes *AtF3H*, *AtCHS*, and *AtCHI* and the regulatory gene *AtPAP1* (Ang et al., 1998; Lee et al., 2007; Shin et al., 2013). In Arabidopsis, AtBBX21 and AtBBX22 can activate transcription. They interact with AtHY5 *in vivo* and induce downstream gene expression in an AtHY5-dependent and -independent manner to promote plant photomorphogenesis (Datta et al., 2007, 2008). In apples and pears, HY5 can bind directly to the *MYB1* promoter (An et al., 2017; Tao et al., 2018). Both PpBBX16 and PpBBX18 cannot bind directly to the *MYB1* promoter, but they can interact with HY5 and promote *MYB1* expression (i.e., HY5-dependent manner) (Bai et al., 2019a,b). However, in apple, MdBBX20 can bind directly to the *MdMYB1* promoter and induce expression (i.e., HY5-independent manner) (Fang et al., 2019b). In this study, we proved that MdBBX21 can bind directly to the *MdMYB1* promoter and induce expression (Figure 8). Additionally, the analysis of protein–protein interactions revealed that MdBBX21 interacts with MdHY5 in yeast and plant cells. Furthermore, the interaction between MdBBX21 and MdHY5 can significantly enhance *MdMYB1* promoter activity (Figure 8).

In conclusion, we systematically characterized the effect of light on MdBBX21 in the apple peel. We proved that MdBBX21 binds to the promoters of *MdBBX20*, *MdBBX22*-*1*/*2*, and *MdHY5*. The subsequent up-regulated expression of these genes enhances anthocyanin accumulation. Additionally, MdBBX21 can interact with MdHY5 and induce *MdMYB1* expression. The results of our study will form the basis of future investigations on the functions of BBX proteins in apple.

## Data Availability Statement

The datasets presented in this study can be found in online repositories. The names of the repository/repositories and accession number(s) can be found below: NCBI SRA BioProject, accession no: PRJNA767632.

## Author Contributions

BZ, H-JY, and Z-YZ conceived the original screening and research plans. BZ, Z-ZZ, B-CW, N-NH, and Y-ZY supervised the experiments. BZ, H-JY, and DQ analyzed the data. BZ and Z-ZZ wrote the manuscript. H-JY revised the manuscript. All authors contributed to the article and approved the submitted version.

## Conflict of Interest

The authors declare that the research was conducted in the absence of any commercial or financial relationships that could be construed as a potential conflict of interest.

## Publisher’s Note

All claims expressed in this article are solely those of the authors and do not necessarily represent those of their affiliated organizations, or those of the publisher, the editors and the reviewers. Any product that may be evaluated in this article, or claim that may be made by its manufacturer, is not guaranteed or endorsed by the publisher.
