# Supplementary material for: MdBBX21, a B-Box Protein, Positively Regulates Light-Induced Anthocyanin Accumulation in Apple Peel
Source: Front Plant Sci. 2021 Nov 12;12:774446. doi: 10.3389/fpls.2021.774446 (PMC8633397; doi:10.3389/fpls.2021.774446)
Supplement: Supplementary file 1 [file Data_Sheet_1.DOCX]

Supplementary Material

**
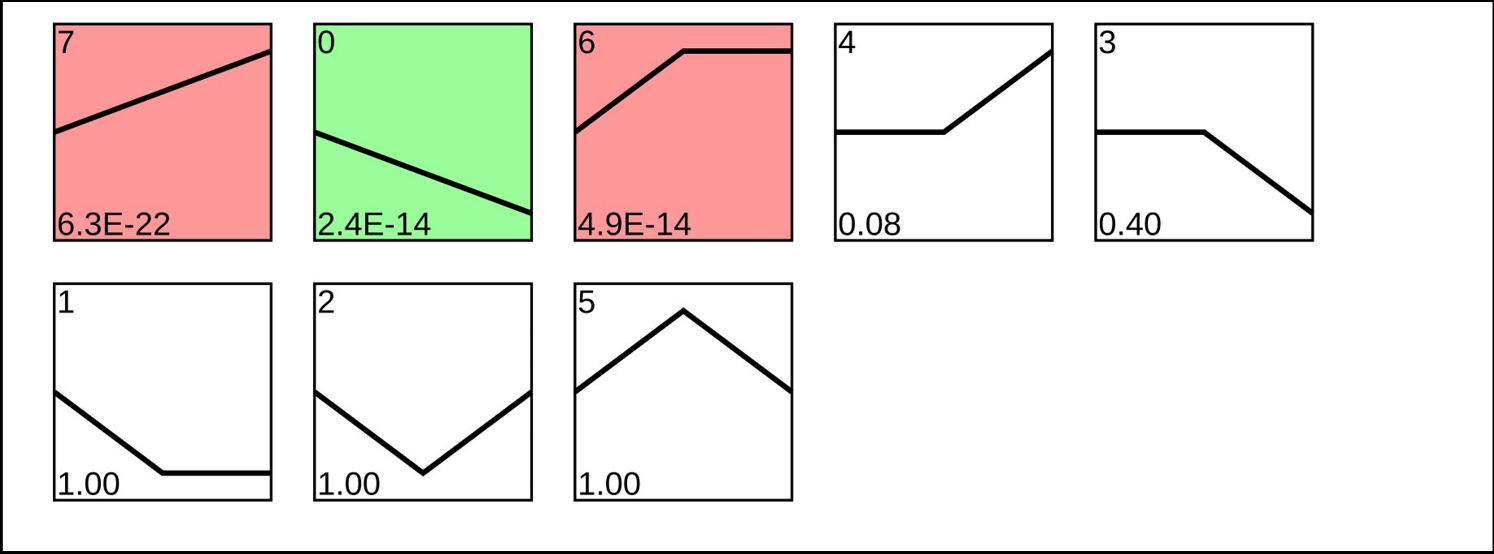
**

**Figure S1.** Profiles of gene expressions across three time points (G0, G6 and G24) by STEM analysis. The black line represented the expression tendency of all the genes.


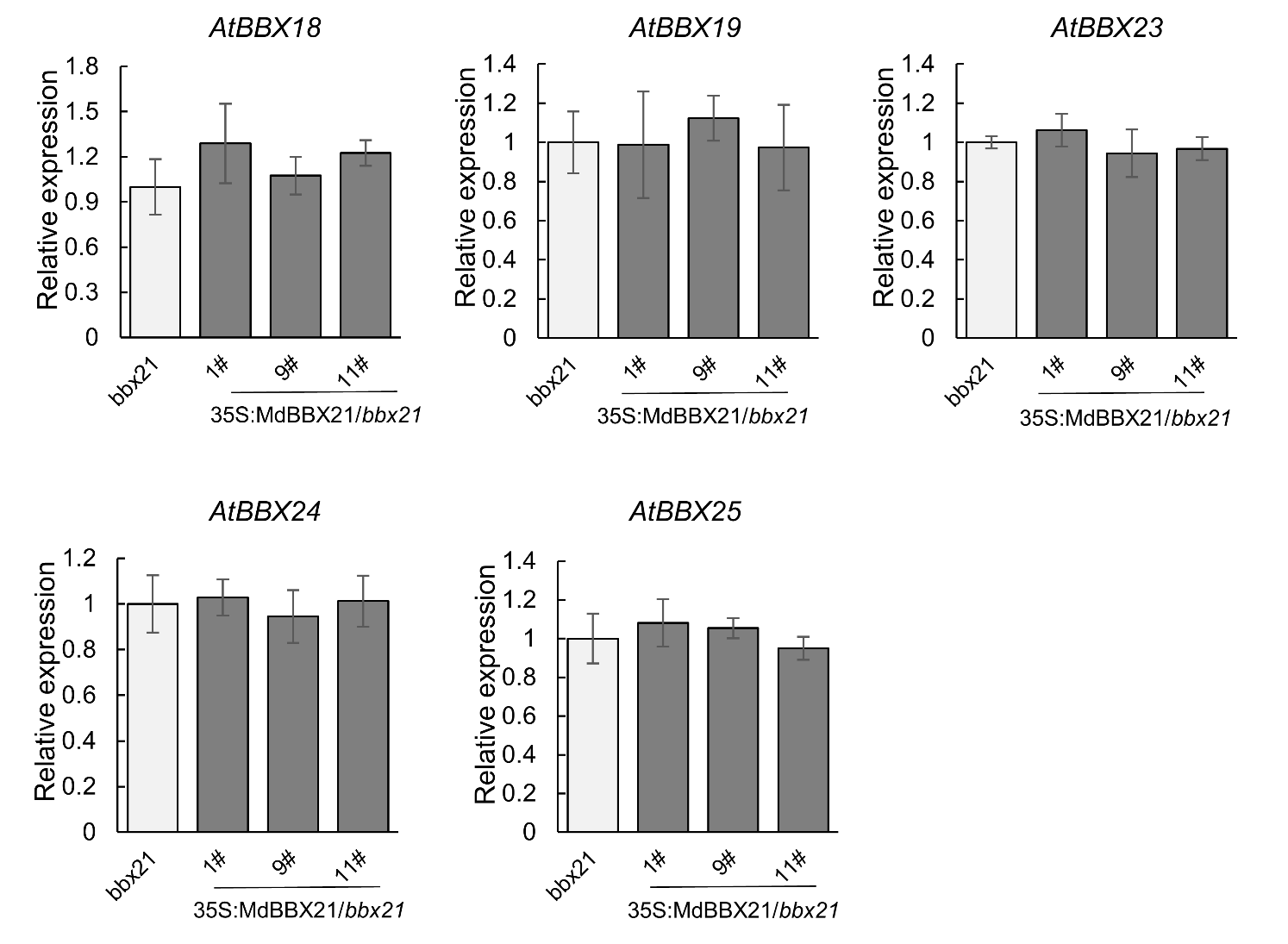


**Figure S2.** Relative expression levels of Arabidopsis subfamily IV members (*AtBBX18*, *AtBBX19*, *AtBBX23*, *AtBBX24* and *AtBBX25*) in 35S:MdBBX21/*bbx21* and *bbx21* mutant seedlings. Error bars represent the standard deviation of three biological replicates.


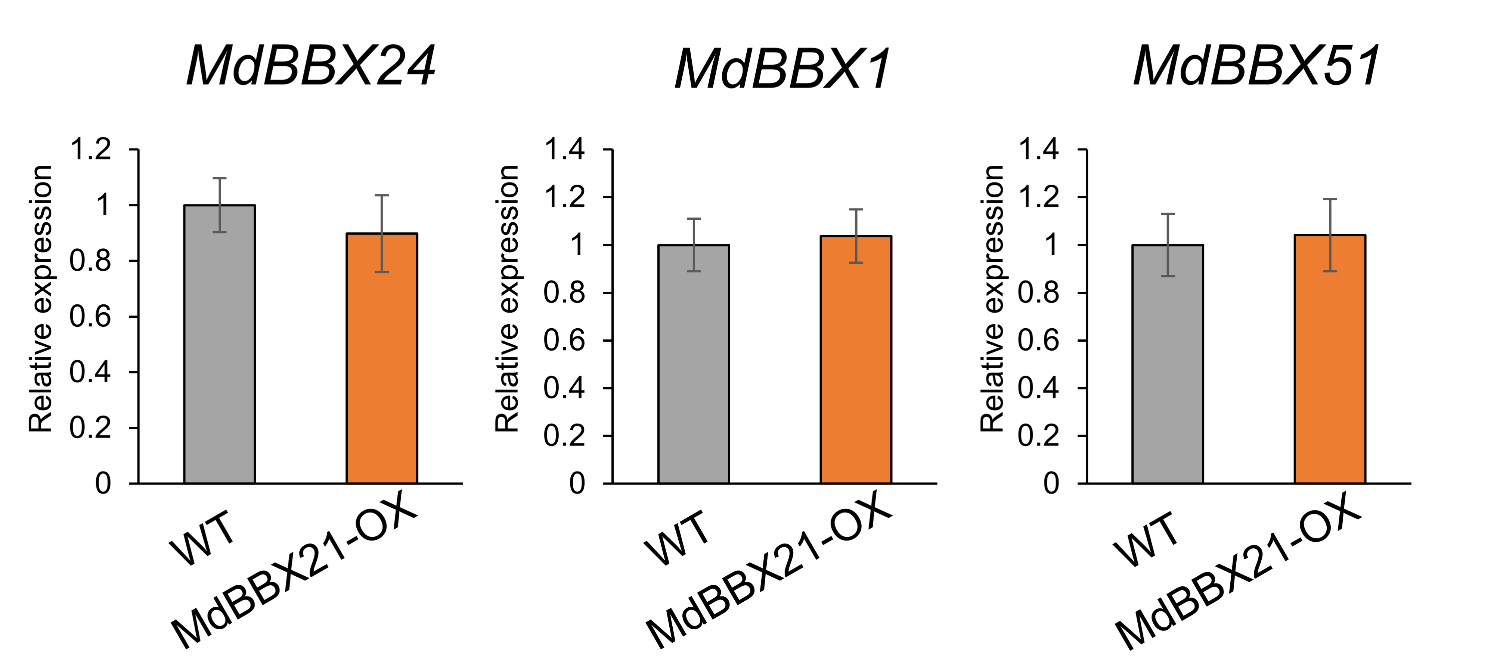


**Figure S3.** The expression levels of *MdBBX24*, *MdBBX1* and *MdBBX51* in WT and MdBBX21-OX. Error bars represent the standard deviation of three biological replicates.


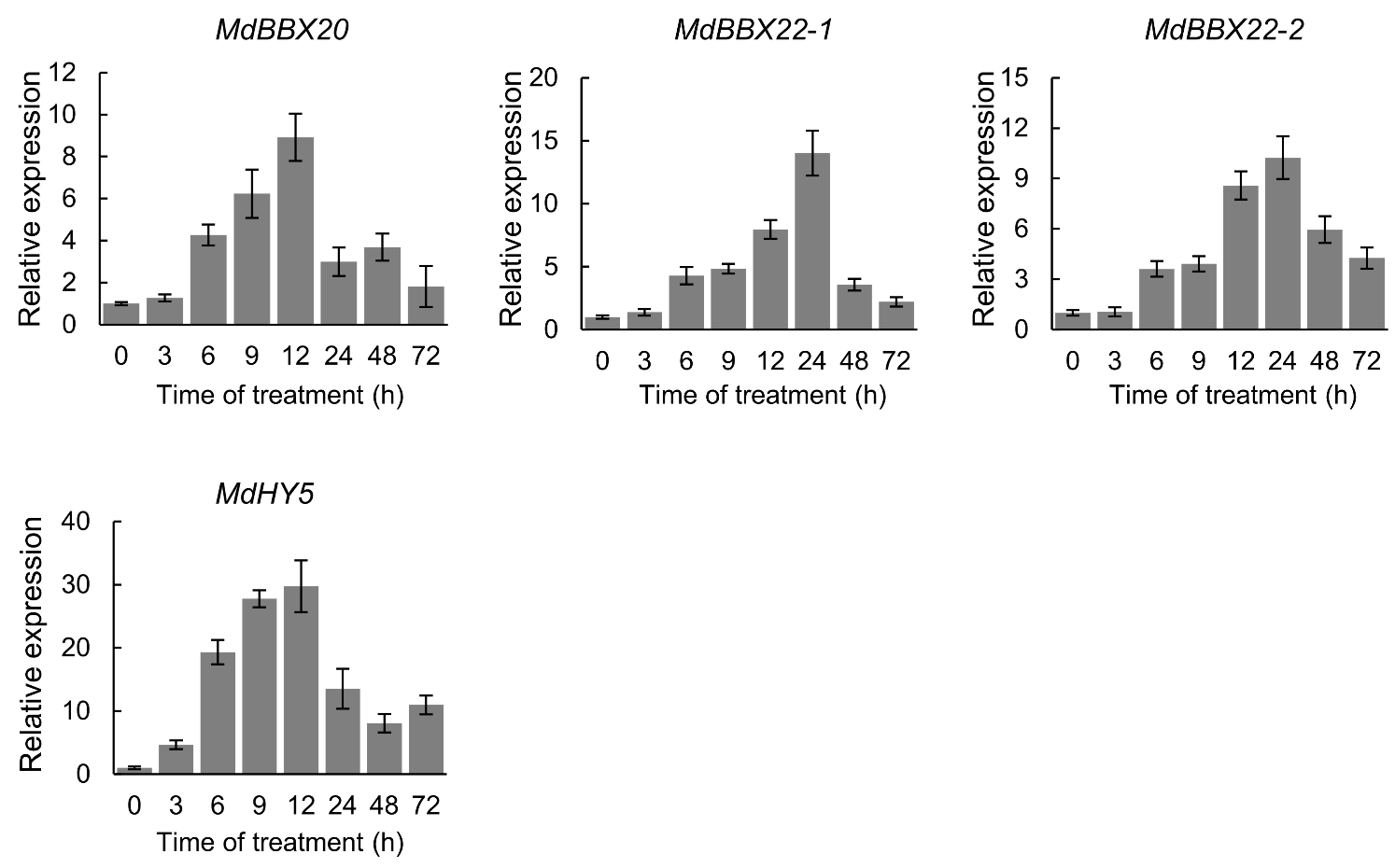


**Figure S4.** Relative expression levels of *MdHY5*, *MdBBX20*, and *MdBBX22*-*1*/*2* in the apple fruit peel irradiated with white light. Error bars represent the standard deviation of three biological replicates.
